# Influence of Baduanjin on lung function, exercise capacity, and quality of life in patients with mild chronic obstructive pulmonary disease

**DOI:** 10.1097/MD.0000000000022134

**Published:** 2020-09-11

**Authors:** Yang Yang, Keling Chen, Wenjun Tang, Xiaohong Xie, Wei Xiao, Jing Xiao, Xialing Luo, Wujun Wang

**Affiliations:** Department of Respiratory Medicine, Hospital of Chengdu University of Traditional Chinese Medicine, Chengdu, P.R. China.

**Keywords:** Baduanjun, mild chronic obstructive pulmonary disease, pulmonary rehabilitation, randomized controlled trial

## Abstract

**Introduction:**

Chronic obstructive pulmonary disease (COPD) is an illness characterized by progressive aggravation of airflow limitation, which seriously affects patients’ quality of life, and even life-threatening. The lung function of COPD patients is chronically and progressively deteriorated. Among them, the lung function of early COPD patients deteriorates rapidly, and forced expiratory volume in 1 second (FEV_1_) declines faster than other stages. If diagnosed early and effectively treated in time, it can greatly affect the prognosis. As a traditional exercise regimen, Baduanjin can improve lung function, exercise capacity, and quality of life of COPD patients. However, high-quality evidence-based medical evidence is so far be lacking to confirm the effectiveness of Baduanjin in reducing or preventing mild COPD lung function decline.

**Methods:**

This study is a randomized controlled trial, 192 patients with mild COPD were randomly divided into experimental group and control group. Both of them will receive basic treatment (health education and Tiotropium bromide), the experimental group will receive Baduanjin exercise training, and the control group will be told to maintain the original lifestyle and control the exercise. The Baduanjin exercise will last for 24 weeks and will be followed up for 72 weeks. The primary outcome is the change in lung function, including FEV_1_, FEV_1_/forced vital capacity (FVC), and FEV_1_/predicted. The secondary results included COPD assessment test, 6-minute walk test, St. George Respiratory Questionnaire, and Dyspnea Scale. Safety will also serve as assessing during the test.

**Discussion:**

The results of this trial will provide that traditional Baduanjin exercises can prevent COPD lung function deterioration, and provide a simple, inexpensive, and daily pulmonary rehabilitation measure for the patients with mild COPD.

## 1 Introduction

Chronic obstructive pulmonary disease (COPD) is an illness characterized by the progressive aggravation of respiratory symptoms and airflow obstruction. Its morbidity and mortality are one of the highest in the world, causing a huge burden on society and the economy.^[1]^ According to estimates by the World Health Organization (WHO), COPD will rise to the third leading cause of death in the world by 2030^[2]^ if no control measures are applied. The global initiative for chronic obstructive pulmonary disease (GOLD) uses the ratio of forced expiratory volume in 1 second to forced vital capacity (FEV_1_/FVC) <0.70 as the diagnostic criteria for airflow obstruction. On this basis, COPD is divided into 4 phases based on the degree of FEV_1_ decline,^[3]^ the proportion of GOLD I and II stages is much higher than that of III and IV.^[4]^ In a retrospective studies, Tantucci and Modina^[5]^ found that the average decline rate of FEV_1_ in GOLD II and III phases was 47 to 79 mL/yr and 56 to 59 mL/yr, respectively, and in GOLD IV, it was lower than 35 mL/yr; Bhatt et al^[6]^ also found that FEV_1_ of COPD patients with GOLD stage I decline fastest, and then gradually slowed down. With the decline of lung function, COPD patients gradually develop to skeletal muscle dysfunction, which affects the patient's athletic ability, reduces the quality of life, and produce anxiety and depression.^[7,8]^ Studies by Burkes and Drummond^[9]^ and Lee et al^[10]^ have shown that early intervention (knowledge education, drug therapy, exercise therapy, etc) for COPD can effectively delay the disease process, improve the mental state of patients, increase exercise tolerance, and reduce social and economic burdens. Therefore, it is necessary to intervene as early as possible.

Pulmonary rehabilitation is an important tool for non-drug treatment of COPD, including but not limited to sports training and education management.^[11]^ Although numerous studies have confirmed that pulmonary rehabilitation can effectively delay the decline of lung function, improve exercise capacity and quality of life in COPD patients.^[12]^ But it has not been fully used due to limitations of venues, equipment, professionals.^[13,14]^ A systematic analysis also indicated a lack of high-quality evidence for the efficacy of traditional pulmonary rehabilitation in mild COPD.^[15]^ Therefore, a simple, easy, safe, and effective pulmonary rehabilitation method for mild COPD is needed. Baduanjin is a traditional Chinese exercise regimen with moderate activity, which can effectively exercise breathing and muscle strength of the limbs, adjust the mental state, and is easy to learn, not restricted by the venue, is very suitable for COPD patients.^[16]^ Previous clinical studies and system analysis have shown that Baduanjin can improve lung function, exercise endurance, and quality of life in COPD patients,^[17–19]^ but it lacks high-quality clinical evidence-based evidence. Therefore, we hypothesized that Baduanjin can delay the decline of lung function, improve exercise ability and quality of life in patients with mild COPD, and designed this experiment for verification.

## 2 Methods/design

### 2.1 Design

**Figure 1:**
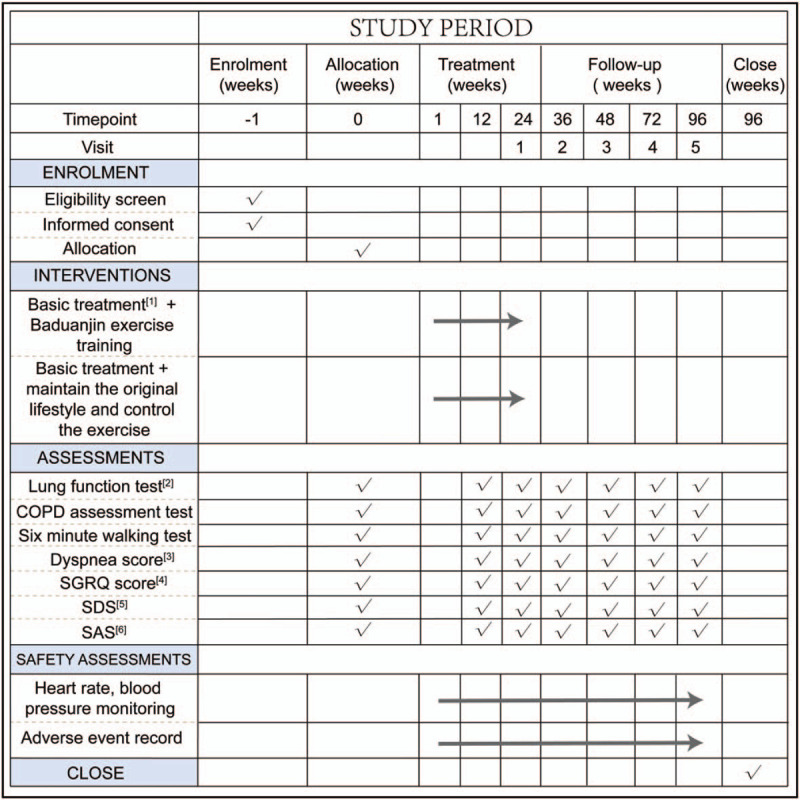
Spirit figure of enrollment, interventions, and assessments. [1] Basic treatment: health education and Tiotropium bromide; [2] lung function test: FEV_1_, FVC, FEV_1_/predicted; [3] Dyspnea score: modified British medical research council dyspnea score; [4] SGRQ score: St. George Respiratory Questionnaire; [5] SDS: Self-rating Depression Scale; [6] SAS: Self-rating Depression Scale.

**Figure 2:**
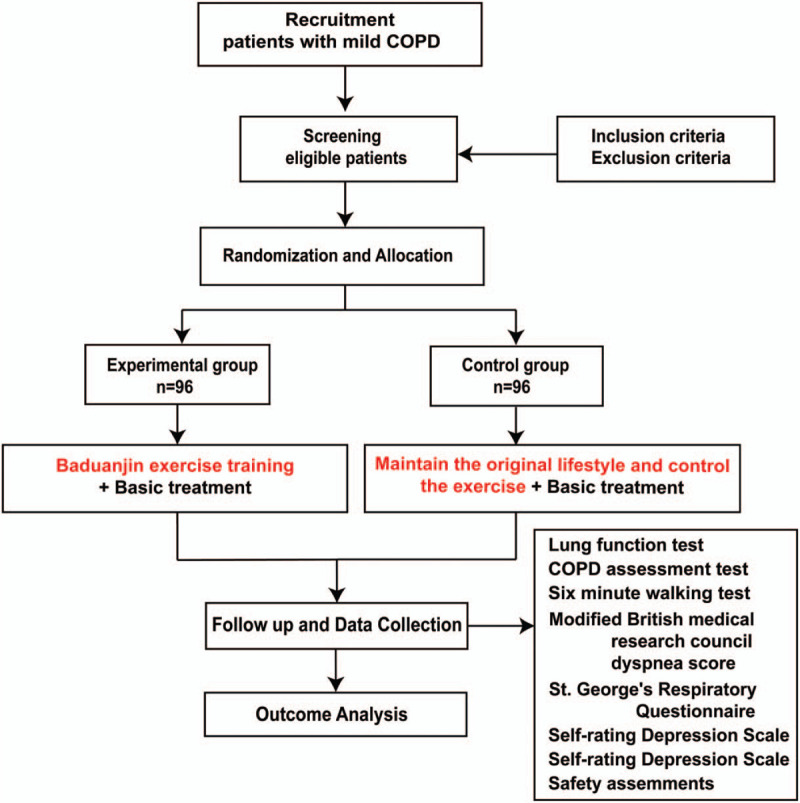
Flowchart of the study design.

This study is a randomized, parallel, and controlled trial designed to evaluate the effectiveness of Baduanjin exercise intervention in improving lung function, exercise capacity, and quality of life in patients with mild COPD. A total of 192 patients will be recruited in Chengdu and randomly assigned to the experimental group or the control group in a 1:1 ratio. Participants in the experimental group will receive a 24-week Baduanjin exercise training intervention at least 5 days a week and 60 minutes a day, while participants in the control group will not be given any specific exercise intervention and will be told to maintain their original lifestyle for 24 weeks. The primary and secondary outcomes will be measured at baseline and at weeks 12, 24, 36, 48, 72, and 96. The participation process of this trial is shown in Fig. 1. This agreement follows the standard agreement item: Intervention Test Recommendation (SPIRIT) guidelines,^[20]^ and meets the SPIRIT list (please see Guidelines Checklist). The study flow chart is presented in Fig. 2.

### 2.2 Ethics approval

The study is in compliance with the Declaration of Helsinki (Edinburgh 2000 versions). The research program has been approved by the Chinese Ethics Review Committee for Registered Clinical Trials (Approval No. ChiECRCT20200189), and the members of the ethics committee tracked the design and implementation of the research. We registered the study in the Chinese Clinical Trial Registry (Registration No. ChiCTR2000035109) in 2020. If there is any amendment to the protocol, approval must be again sought from the Ethics Committee.

### 2.3 Recruitment

Participants will be recruited through local advertisements and doctors from the Department of Respiratory Medicine, Affiliated Hospital of Chengdu University of Traditional Chinese Medicine. Before recruiting, participants will be provided with details about the clinical study, including its purpose, grouping methods, the differences between the experimental group and the control group, study schedule, and possible risks and benefits. Recruiters will explain to patients that the basic treatments of the 2 groups are according to the guidelines recommended by the GOLD^[3]^ and the “Guidelines for the Diagnosis and Treatment of Chronic Obstructive Pulmonary Disease (2013 Revised Edition),”^[21]^ and that Baduanjin exercise training is a safe and effective pulmonary rehabilitation measure to ensure that subjects can receive randomization and in strict accordance with the protocol. All patients will be required to sign informed written consent prior to beginning any research procedure.

### 2.4 Sample size

This study is a randomized controlled trial. The experimental group is basic treatment (health education and Tiotropium bromide) + Baduanjin exercise training, and the control group is basic treatment + maintain the original lifestyle and control the exercise. Pulmonary function (FEV_1_%) is the main outcome index. According to previous literature.^[19]^ The mean and standard deviation of FEV_1_% of the control group was 57.09 and 22.53, while the mean and standard deviation of FEV_1_% of the experimental group was 60.24 and 20.15, set *α* = 0.05 (bilateral), *β* = 0.10. The sample size N1 = N2 = 77 cases of experimental group and control group were calculated by Power Analysis and Sample Size version 15.0.5 (PASS 15.0.5 NCSS, LLC USA). Assuming a 20% dropout rate for the study subjects, 96 samples were needed. Therefore, the lowest sample size was 192.

### 2.5 Randomization and allocation concealment

Members of the Sichuan Evidence-based Medicine Center of Traditional Chinese Medicine will use SAS 9.2 software (SAS, Cary, NC) to generate 192 random serial numbers. The grouping and treatment plan will be placed in an envelope made of carbon-free carbon paper and numbered in random serial numbers. And will be kept by an administrator who is not involved in recruitment and subsequent experiments. After signing the informed consent and receiving the baseline assessment of the primary and secondary outcome measures, the administrator will open the envelope in turn and provide the group number to the eligible participants. Finally, all participants will be randomly divided into the experimental group or the control group at a ratio of 1:1.

### 2.6 Blinding

Due to the particularity of sports training, the participants, coaches, and follow-up personnel cannot be blinded, we will blind the result assessors through the following methods to ensure the reliability of the results. The results will be evaluated by a new person not involved in the previous experiment. Participants, sports coaches, and research assistants will be advised not to disclose any information about the assignment before the outcome evaluation begins. Therefore, the outcome evaluator will not know the assignment of the protocol until the end of the study.

### 2.7 Diagnostic criteria

All participants must meet the Western medicine diagnostic criteria for COPD^[3]^ (Table 1) and Classification of airflow limitation severity in COPD (based on post-bronchodilator FEV_1_)^[3]^ (Table 2).

**Table 1:**
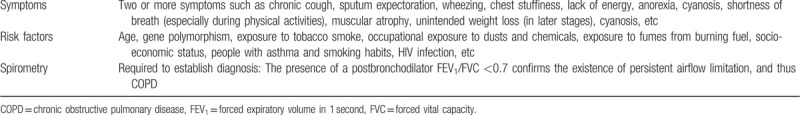
Diagnostic criteria for chronic obstructive pulmonary disease^[3]^.

**Table 2:**
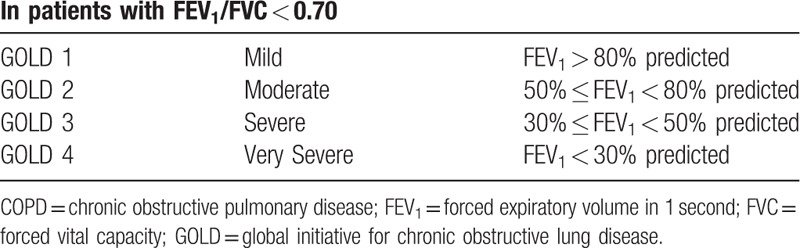
Classification of airflow limitation severity in COPD (based on post-bronchodilator FEV_1_)^[3]^.

### 2.8 Eligibility criteria

Inclusion criteria:

(1) Age range 40 to 70 years, men or women;
(2) Mild COPD defined by GOLD guidelines: FEV_1_/FVC <70% and FEV_1_ ≥80% predicted value;
(3) In the stable phase of COPD, no respiratory infections and acute exacerbations of COPD in the past 4 weeks;
(4) Have not performed Baduanjin and similar Qigong exercises in the past;
(5) Volunteer to take part in research and provide informed consent.

Exclusion criteria:

(1) Patients with chronic lung diseases (such as bronchial asthma, interstitial lung disease, active tuberculosis, or bronchiectasis) need intervention or treatment;
(2) Patients suffering from severe primary diseases (such as severe mental illness) diseases, cardiovascular and cerebrovascular diseases, liver and kidney diseases, endocrine diseases, hematopoietic diseases, malignant tumors, etc;
(3) Patients with limited physical activity or other reasons who cannot perform Baduanjin training;
(4) The patient has participated in any other clinical studies in the past 6 months.

### 2.9 Termination and withdrawal criteria

All participants will be informed of their right to withdraw from the trial and, if so, will receive standardized treatment. The reasons for the withdrawal will be recorded in their case report file (CRF). The criteria for stopping treatment and pulling patients out of the study are:

- Participants have experienced exercise-related adverse events, and the researchers believed that they are unfit to exercise;
- Participant has another serious illness and needs to be treated during the study;
- The participant has suffered a severe exacerbation;
- Poor compliance so that exercise time is <80% of the prescribed time;
- Perform other pulmonary rehabilitation exercises during the study.

## 3 Interventions

### 3.1 Treatment plan

Both the experimental group and the treatment group will according to the guidelines recommended by “Global strategy for prevention, diagnosis and management of chronic obstructive pulmonary disease (GOLD)”^[3]^ and the “Guidelines for the Diagnosis and Treatment of Chronic Obstructive Pulmonary Disease (2013 Revised Edition)”^[21]^:

Health education: education and urge quit smoking; to understand the pathophysiology and clinical basic knowledge of COPD; to understand the opportunity to seek medical treatment in hospitals; vaccination; to prevent colds and avoid dust, smoke, and harmful gases.

Medication: Tiotropium bromide powder inhalation (Silihua, Boehringer Ingelheim Pharma GmbH & amp; Co.KG [Germany]) 18 μg, inhaled by inhaler once a day.

#### 3.1.1 Experimental group

In addition to basic treatment (Health education and Tiotropium bromide), Baduanjin training is also performed. The first week will be supervised and guided by professionals from Chengdu University of Traditional Chinese Medicine to teach Baduanjin (version from the General Administration of Sports). At least 5 days a week, 60 minutes for each training, which can be divided into 30 minutes in the morning and afternoon according to personal tolerance. After the first 2 weeks, the patients will train at home, and they will be given Baduanjin Tutorial CD and exercise card book to record the daily training. From the 3rd week, there will be an intensive training every Sunday. Two professionals will review the record of the exercise card and guide corrective actions until the end of 24 weeks.

#### 3.1.2 Control group

The control group members will not receive any training while undergoing basic treatment, and they will be told to maintain the original lifestyle and control the amount of exercise. At the same time, each participant will be issued a daily activity record card to record the number of daily WeChat steps and additional activities.

### 3.2 Outcome measures

Primary outcome: The primary outcome is the change in lung function from baseline to the end of the follow-up period (all time points). Including FEV_1_, FVC, FEV_1_/predicted value.

Secondary outcomes: Including COPD assessment test (CAT), 6 minute walking test (6MWT), dyspnea score (modified British medical research council dyspnea score, mMRC), and St. George Respiratory Questionnaire (SGRQ), Self-rating Depression Scale (SDS), Self-rating Depression Scale (SAS).

### 3.3 Compliance

In this trial, compliance was quantified by participation in collective training and exercise book recording. The researchers will make every reasonable effort to follow the patients during the study. Messages will be sent via WeChat or over the phone at each training and follow-up session. Patients with a total time of >80% recorded in the collective training and exercise card will be considered as compliant.

### 3.4 Safety assessment and adverse events

During each intensive exercise, we will monitor the heart rate and blood pressure, and ask for details of adverse events such as muscle strain, falls, dizziness, dyspnea, psychological pressure, or COPD exacerbation, etc. For any symptoms identified, we will further document their relevance to the intervention, determine the required changes in treatment depending on severity, and provide further medical care. For hospitalization and emergency visits, we further determined the outcome of the discharge diagnosis and the main cardiopulmonary examination or procedure performed. During the intervention process, any adverse events will be recorded in the CRF and reported to the Steering Committee and the Ethics Committee within 24 hours. If any severe adverse events occurring, training will be discontinued, and reported to the Ethics Committee and receive appropriate treatment.

### 3.5 Data management and quality control

All records will be collected in the CRF and completed by trained and qualified investigators. After the CRF is accomplished, if any corrections are made, the original record will not be changed. The clinical inspector will review the completed CRF. Data input and management will be carried out under the guidance of medical statistics experts. In order to guarantee the accuracy of the data, two data administrators will input and proofread the data separately. After checking and confirming that the established database is correct, the data will be locked by the main researchers and statistical analysts. Locked data or files will not be changed from now on and will be submitted to the research team for statistical analysis. Sichuan Evidence-based Medicine Center of Traditional Chinese Medicine (Chengdu, China), which has no conflicts of interest, will monitor the data. The scientific research room of Chengdu University of Traditional Chinese Medicine Hospital, which is independent of researchers, will conduct data review during the experiment.

### 3.6 Statistical analysis

All data analysis will be carried out in accordance with the principle of intentional processing. The missing value will be substituted for the last observation carried forward method. Before the analysis, 2 similar participants with complete data will be carefully checked to ensure that the data are correct.

The data will be analyzed using a social science version 22.0 statistical software packages (SPSS 22.0, Chicago, IL). The analysis method will be selected according to the distribution characteristics of the data: the measurement data will be checked using group *t* test or non-parametric test, the count data will be tested using chi-square test or Fisher exact probability method, and the score data will be tested using non-parametric test. Compared with the baseline value, paired *t* test or non-parametric test will be used to evaluate measurement data, and non-parametric test will be utilized to test count data. All statistical tests will be bilateral tests, and a *P* value <.05 will be deemed to indicate statistical significance.

## 4 Discussion

COPD is a major public health problem that seriously affects the quality of life of patients, even life-threatening. The sign of COPD progression is decreased lung function, and pulmonary rehabilitation has been proven to be the most effective treatment strategy for improving shortness of breath, health status, and quality of life.^[13]^ However, traditional pulmonary rehabilitation requires a series of professional evaluations to develop a perfect and personalized plan,^[22]^ so the overall effect is not satisfactory, and it is urgent to develop family-based pulmonary rehabilitation methods.^[23]^ Baduanjin is the representative of the traditional Chinese medicine guidance method, covering endurance training, breathing muscle training, stretching training, and psychological adjustment required for pulmonary rehabilitation. The whole set of movements is easy to learn, moderate intensity, and significant efficacy, which applicability and cost are much better than traditional pulmonary rehabilitation. However, previous studies have mostly focused on the efficacy of Baduanjin in the middle and late stages of COPD, and there is no unified understanding of its intervention effect in mild COPD pulmonary function decline. Therefore, this study focuses on the effect of Baduanjin on lung function in the early stage of COPD.

As far as we know, this is the first clinical study on the treatment of mild COPD with Baduanjin exercise training. In our research, we will use validated objective tools such as lung function test, COPD assessment test, 6-minute walk test, etc. These measurements improve the reliability and versatility of the results. This study has limitations. It is difficult to monitor physical activities outside the interventions of the experimental group and the control group. Although all participants are required to maintain the original lifestyle and exercise volume, this is not enough to limit activities that may affect the experimental results. Baduanjin belongs to traditional Chinese guiding Qigong, and it is unclear whether its exercise mode and intensity are suitable for people in other regions. The condition of mild COPD is related to many factors, and the influence of education management and conventional drug treatment on the efficacy of Baduanjin cannot be excluded. Despite the limitations, we believe that this study will help discover the benefits of Baduanjin in preventing lung function decline, improving exercise performance, and improving quality of life in patients with mild COPD. In the future, multicenter randomized controlled trial (RCT) and multi-dimensional comprehensive comparison should be performed.

## Acknowledgments

The authors are grateful to the Sichuan Science and Technology Program for funding this study. They also would like to thank Editage (www.editage.cn) for English language editing.

## Author contributions

**Conceptualization:** Yang Yang, Keling Chen, Wenjun Tang.

**Investigation:** Wujun Wang, Jing Xiao.

**Supervision:** Xiaohong Xie, Wei Xiao.

**Writing – original draft:** Yang Yang.

**Writing – review & editing:** Xialing Luo, Wujun Wang.
